# Editorial Notes, News and Miscellany

**Published:** 1914-06

**Authors:** 


### Editorial Notes, News and Miscellany.

DR. J. C. THOMAS announces to his friends and patrons that he is now located at Taylor, Texas, and will confine his work to surgery, gynecology and consultations.

---

DR. H. S. BERNSTEIN, director of the Bender Hygienic Laboratory, Albany, New York, has been appointed pathologist and bacteriologist of the Rhode Island Board of Health at a salary of $4000 a year.

---

WE NOTE with pleasure that at its recent annual convocation, June 3, 1914, the Hamilton College of Law of Chicago conferred upon our friend, Dr. G. Frank Lydston, the degree of Doctor of Civil Laws.

---

DR. FRANK PASCHAL, of San Antonio, has contributed to the TEXAS MEDICAL JOURNAL an able article on "The Necessity for Centralization of Power for Protecting Public Health," which will appear in the July number.

---

DR. JAMES G. KIERMAN, of Chicago, in an early issue of the JOURNAL will discuss the "Cumulative Effects of Hypnotics." This theme will be of much interest to all physicians, as it is a subject that is attracting much attention both of the physician and laity.

---

IN THE NEXT NUMBER of the "Red Back," Dr. Morris Boerner of hookworm fame, will tell us something of the progress of the work in the State. Dr. Boerner is giving his entire time and attention to the study of this subject, and his article will be interesting and helpful.

---

THE SECOND ANNUAL CONVOCATION of the American College of Surgeons will be held Monday, June 22d, at the Bellevue-Stratford Hotel, Philadelphia. It is expected that from 500 to 700 additional fellowships will be conferred upon applicants who have been accepted since the last convocation.

---

MANY OF THE CITIES of the country are having "Baby Weeks" to interest everyone in the public and private care of babies in such a way as to reduce the infant mortality rate of the country. In the last few years the death rate of infants in this country has fallen from 135.8 per 1000 to 101.9 per 1000.

---

FOR THE SESSION of the American Medical Association to be held at Atlantic City, June 22d to 26th, a most attractive program has been prepared. The railroad rate from Missouri river points will be approximately $43 for the round trip, with return limit of thirty days. The rates from other sections are proportionately low. It is expected that an unusually large number of physicians will attend from Texas and the Southwest. The Rudolf Hotel, Atlantic City, will be headquarters for Southwestern physicians.

---

THE General Board of Education has made a grant of $500,000 to Yale as an endowment for the medical school, it being a condition of the grant that the school shall procure complete teaching and medical control of New Haven Hospital, and that the teachers in the main clinical branches be placed on a full-time or university basis. The General Board of Education has under consideration the establishment of schools for the training of public health officers in one or two Eastern centers like Boston and New York.

---

AMERICAN MEDICAL EDITORS.—An attractive program has been arranged for the American Medical Editors at Atlantic City, N. J., on the opening day of the American Medical Association's session, June 22d. Among the papers are the following:

President's Address, E. A. Vander Veer, M. D., Albany, N. Y.; "Relation of the Medical Press to the Cancer Problem," by Mr. Frederick L. Hoffman, Statistician of the Prudential Insurance Co., Newark, N. J. (by invitation); "The Things That Count in Medical Practice," by H. Edwin Lewis, M. D., New York; "Ideal National Medical Journal; What It Should Be and What It Should Not Be," by W. J. Robinson, M. D., New York; "Two Problems of the Organization Journal; the Mediocre Paper and the Editorial Department," by Sarah M. Hobson, M. D., Chicago, Ill.; "Medical Journalism as a Local and as a National Proposition," by Thomas S. Blair, M. D., Harrisburg, Pa.; "Medical Books and Journals," by T. D. Crothers, M. D., Hartford, Conn.; "The Medical Periodical and the Scientific Society," by F. H. Garrison, M. D., Washington, D. C.; "Editorial Experiences," by A. L. Benedict, M. D., Buffalo, N. Y.; "The Special Medical

Journal," by A. Bassler, M. D., New York; "The Medical Profession and Its Influence from a Buying Standpoint," by Joseph MacDonald, Jr., M. D., New York; "The Preparation of the Original Article and the Editor's Latitude," by E. Franklin Smith, M. D., New York; "He Among You Who Is Without Sin Shall Cast the First Stone," by Erwin Reissmann, M. D., Newark.

